# Supplementary material for: Comprehensive analysis of yeast +1 ribosomal frameshifting unveils a novel stimulator supporting two distinct frameshifting mechanisms
Source: Nucleic Acids Res. 2025 Nov 28;53(22):gkaf1301. doi: 10.1093/nar/gkaf1301 (PMC12661321; doi:10.1093/nar/gkaf1301)
Supplement: gkaf1301_Supplemental_File [file gkaf1301_supplemental_file.pdf]

Supplementary material for

Comprehensive analysis of yeast +1 ribosomal frameshifting unveils a novel stimulator affirming two distinct frameshifting mechanisms

Darren A Fenton[1], Maria Bożko[2], Michał Świrski[2], Gary Loughran[1], Martina M Yordanova[1], Joanna Kufel[2], John F Atkins[1,3], Pavel V Baranov[1*]

[1]School of Biochemistry and Cell Biology, University College Cork, Ireland
[2]Institute of Genetics and Biotechnology, Faculty of Biology, University of Warsaw, Warsaw, Poland
[3]School of Microbiology, University College Cork, Cork T12 K8AF, Ireland.
*Corresponding author: p.baranov@ucc.ie

Table of Content:

Supplementary Figure 1. 2
Supplementary Figure 2 3
Supplementary Figure 3 4

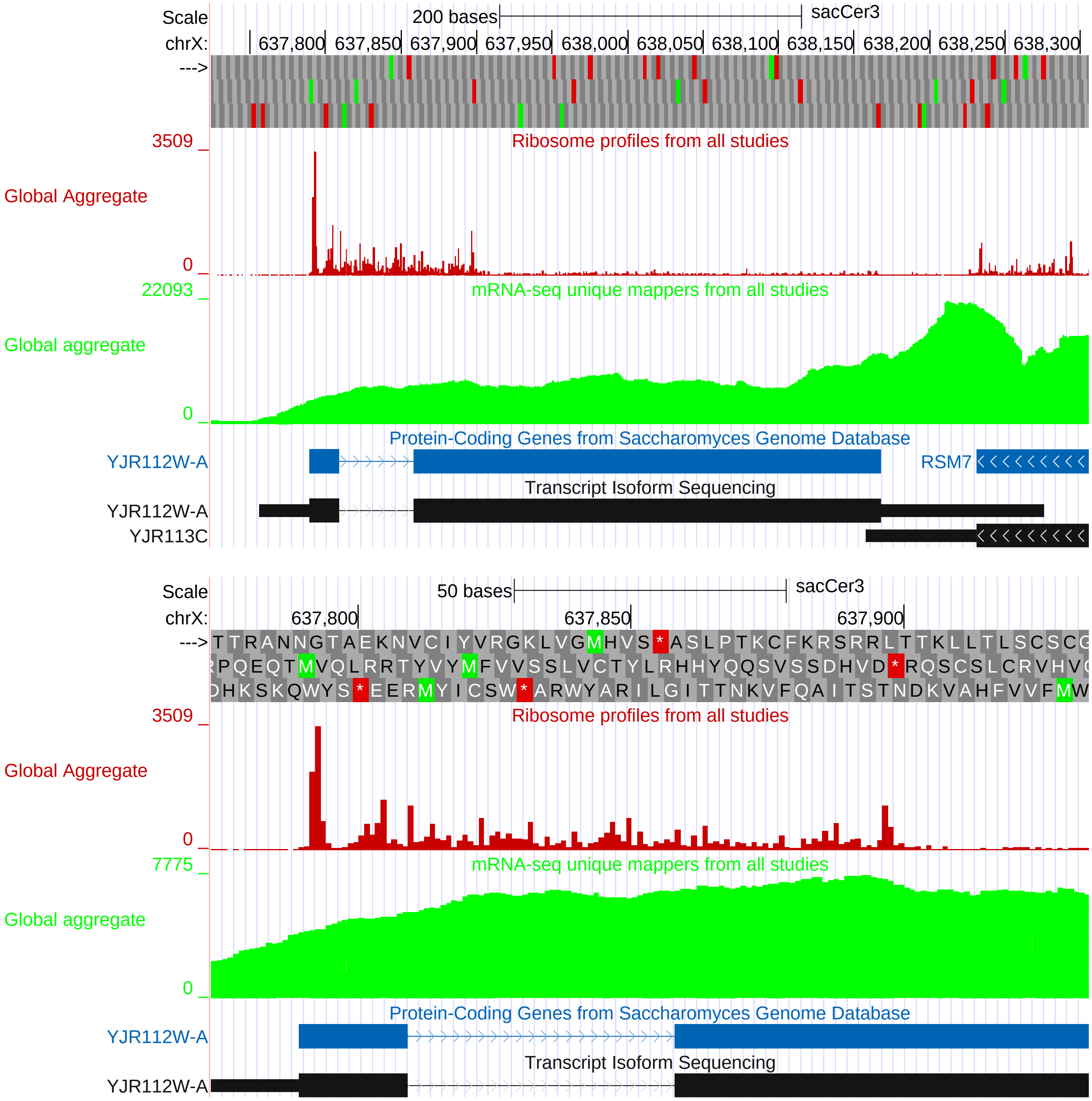

Supplementary Figure 1. GWIPS-Viz snapshot of the *S. cerevisiae* YJR112W-A locus (Top). Global aggregate track of Ribo-seq data is presented as red bars reflecting A-site positioning of the translating ribosomes. RNA-seq coverage is presented as the lime (green) track. Bottom panel is the same as top, but with a zoom and focus on the 0-frame ORF that contains the +1 frameshifting sequence CUU_A.GG_C. Note the incorrect annotation tracks that suggest an intronic sequence in the 0-frame ORF.

Kluyveromyces marxianus

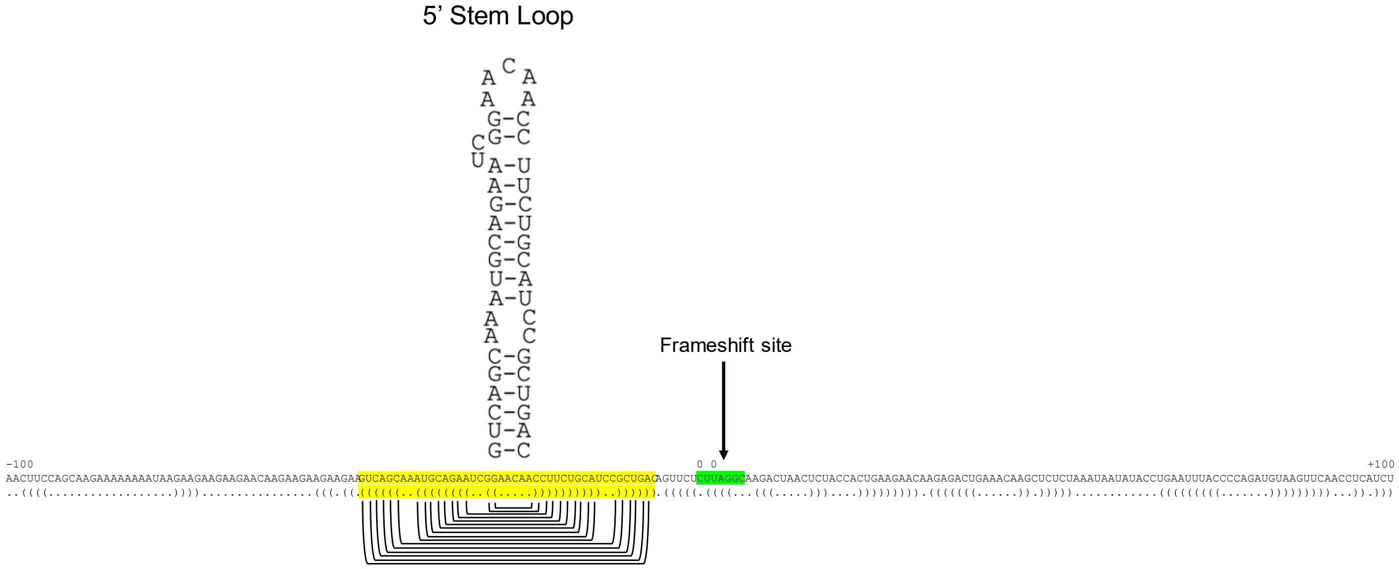

Supplementary Figure 2. Sequence of the *Kluyveromyces marxianus* ABP140 mRNA including frameshiftong heptamer (in green) and the 100 nt upstream and downstream. The stem loop located 6 nt upstream from the frameshift site is highlighted in yellow. The predicted secondary structure is indicated below in dot and bracket format, a diagram of of the stimulator's secondary structure is also shown above.

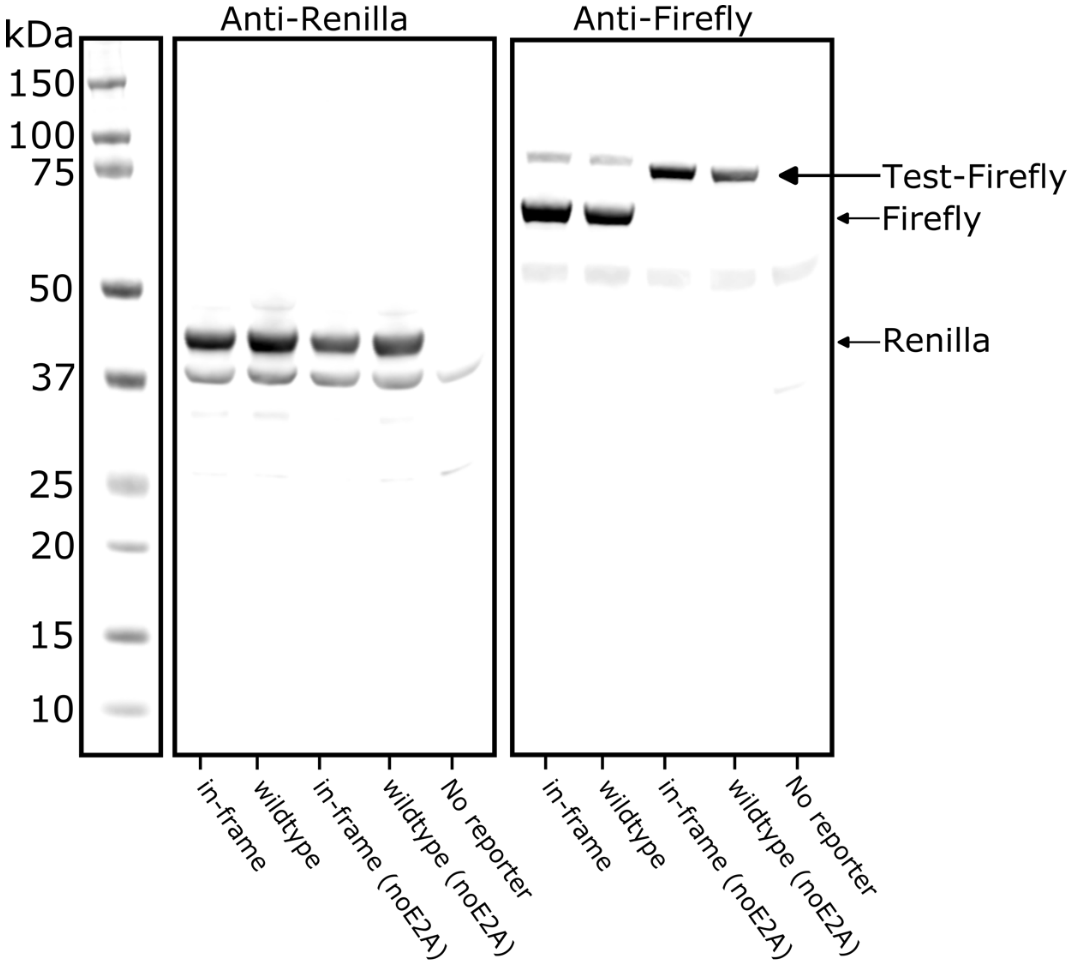

Supplementary Figure 3. Western blotting of yeast protein extracts from the cells expressing reporter mRNAs containing ABP140 ribosomal frameshifting cassette and controls. Western blotting was performed with anti-Renilla and anti-Firefly antibodies. As a negative control, yeast cells expressing no plasmid was used. Note that in the noE2A reporter, the downstream (or second) StopGo sequence has been deleted. “Test-Firefly” bands represent the protein products when the second E2A is removed.
